# Isolated Unilateral Optic Neuritis Secondary to Presumed Ocular Tuberculosis in an Immunocompetent Adult

**DOI:** 10.7759/cureus.111725

**Published:** 2026-06-29

**Authors:** Preshaantini Ponnaiah, Chandramalar T. Santhirathelagan, Shamala Retnasabapathy, Wan Haslina Wan Abdul Halim

**Affiliations:** 1 Ophthalmology, Universiti Kebangsaan Malaysia, Hospital Sungai Buloh, Sungai Buloh, MYS; 2 Ophthalmology, Hospital Sungai Buloh, Sungai Buloh, MYS; 3 Ophthalmology, Universiti Kebangsaan Malaysia Medical Centre, Kuala Lumpur, MYS

**Keywords:** anti-tb, extra pulmonary, ocular tuberculosis, optic neuritis, tuberculosis

## Abstract

This case report describes tuberculous optic neuritis as the presenting feature of tuberculosis (TB) in an immunocompetent adult. A 23-year-old inmate presented with four days of painless left eye vision loss without systemic TB symptoms. Visual acuity (VA) was hand movement with relative afferent pupillary defect. Left eye light brightness was reduced, but no red desaturation. Funduscopy showed a hyperaemic optic disc and blurred disc margin, with dilated, tortuous vessels. Visual field revealed a central scotoma. Systemic examination was unremarkable. Investigation showed a normal chest X-ray, raised erythrocyte sedimentation rate, positive Mantoux, and TB Quantiferon. Lumbar puncture revealed normal opening pressure and biochemistry with negative cultures. Cerebrospinal fluid electrophoresis demonstrated oligoclonal bands, suggesting inflammation/infection. Magnetic resonance imaging showed a slightly bulky left optic nerve with T2 hyperintensity and post-contrast enhancement, suggesting optic neuritis. Anti-TB treatment improved vision to 6/6 with normal optic nerve function and a well-defined optic disc margin. Tuberculous optic neuritis requires a high index of suspicion, as corticosteroids, which are the standard treatment for optic neuritis, may worsen the outcome without anti-tubercular treatment.

## Introduction

Tuberculosis (TB) is caused by *Mycobacterium tuberculosis* (MTB) and is an airborne infectious disease transmitted through inhalation of infected respiratory droplets. According to the World Health Organisation (WHO), globally, TB is the major cause of death from a single infectious agent. It has been reported that over 10 million people contract TB annually, and more than one million lose their lives to this disease [1].

TB is a preventable and curable disease. The mortality rate of TB is about 50% when no treatment is initiated; however, with the current WHO recommended treatment regimen of anti-TB drugs for four to six months, 90% of those infected with TB can be cured [1].

In the WHO Global Tuberculosis Report 2025, 30 countries are identified as high TB burden countries. Although Malaysia is not classified as one of them, it neighbours several high-burden countries, such as Indonesia, the Philippines, and Myanmar. Notably, Indonesia is among the top eight countries with a high TB burden globally [1]. The incidence rate of TB in Malaysia is about 50-99 cases per 100 000 population, classifying it as an upper-moderate TB burden country [2]. TB is frequently associated with poverty. Individuals with low socio-economic status are more likely to have cramped and poorly ventilated living and working conditions, greater food insecurity, reduced awareness of health issues, and limited access to health care, which are all risk factors for the spread of this disease [2].

TB is most commonly associated with pulmonary infection. However, it can also affect other organs, including the eye. Ocular TB is rare and only accounts for about 1-2% of all TB cases [3] and 3.5-5.1% of all extrapulmonary TB cases [4]. Although rare, ocular TB often causes a significant impact on visual function and affects the quality of life of those affected by the disease [4].

Intraocular manifestations of TB can mimic various other diseases, and it has a chronic clinical course. This report presents a case of isolated unilateral optic neuritis secondary to TB in an immunocompetent adult.

## Case presentation

A healthy 23-year-old man who is an inmate presented with left eye painless central vision loss for four days duration. There was no history of eye discomfort, eye pain on ocular movement, eye redness, or double vision. He had no significant past ocular trauma or surgery, and he denied symptoms of a systemic tuberculosis infection, such as prolonged cough, haemoptysis, fever, night sweats, loss of weight, or poor appetite. He also denied any drug usage.

On examination, his left eye visual acuity (VA) was hand movement with a relative afferent pupillary defect. The anterior segment was unremarkable. Funduscopy showed a left eye hyperaemic optic disc, a blurred disc margin nasally, with dilated and tortuous inferior quadrant retinal vessels. The retina and macula were otherwise normal (Figure 1B). The right eye examination was unremarkable with unaided VA of 6/9 (Figure 1A).

**Figure 1:**
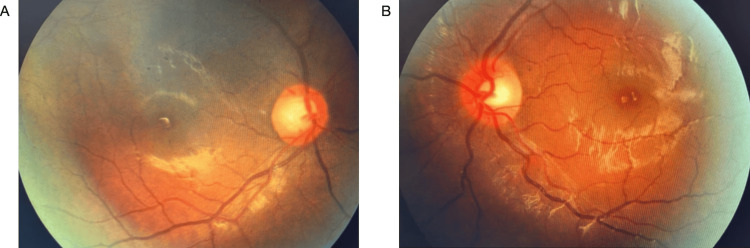
(A) Normal right optic disc and retinal vessels. (B) Left optic disc hyperaemia with a blurred disc margin nasally and dilated, tortuous inferior quadrant retinal vessels.

Visual field testing in the left eye revealed a central scotoma, and the right eye visual field was normal. On general examination, he was alert and oriented well to time, place, and person. His lungs were clear, and he had no lymphadenopathy. His central nervous system examination was also normal.

Haematological investigations revealed a raised erythrocyte sedimentation rate (ESR) of 28 mm/hour. The other tests that were positive were the Mantoux test, which showed a 20-mm area of induration, and the TB Quantiferon test. Autoimmune, inflammatory, and infective screening were negative. Lumbar puncture showed a normal opening pressure of 15 cmH_2_O, normal biochemistry analysis, and negative cultures. Cerebrospinal fluid (CSF) MTB gene expert was also not detected (Table 1).

**Table 1:** Summary of relevant investigations. ESR: Erythrocyte Sedimentation Rate; VDRL: Venereal Disease Research Laboratory; C&S: Culture and Sensitivity; MTB: *Mycobacterium tuberculosis*

Parameters	Patient value	Reference range
ESR	28 mm/hour	0-15 mm/hour
TB Quantiferon	Positive	
Rheumatoid factor	<4 IU/mL	0-14 IU/mL
Complement C3	1.34 g/L	0.96-1.74 g/L
Complement C4	0.47 g/L	0.19-0.47 g/L
Proteinase 3 antibody	Negative	Negative if <2.3
Myeloperoxidase	Negative	Negative if <3.2
Antinuclear antibody	Negative	
HIV, hepatitis B, hepatitis C, syphilis serology	Non-reactive	
Leptospira IgM	Negative	
Bartonella Henselae and Bartonella Quintana IgG and IgM	Negative	
Burkholderia pseudomallei IgM	Negative	
Cerebrospinal fluid analysis		
Glucose	3.2 mmol/L	2.2-3.9 mmol/L
Protein	0.41 g/L	0.15-0.45 g/L
Cell count (red blood cell and white blood cell)	0	
Gram stain	No pus cells, no organisms	
Indian ink	No encapsulated yeast	
VDRL	Non-reactive	
C&S	No growth	
MTB C&S	No AFB seen, no growth	
MTB Gene Expert	Not detected	

Both CSF and serum electrophoresis showed the presence of a similar pattern of oligoclonal bands, suggestive of systemic inflammation/infection. However, the albumin and IgG quantification could not be done due to an insufficient CSF sample.

Chest X-ray was reported to be normal. The MRI of the brain, orbit, and spine showed a slightly bulky retrobulbar intraorbital segment of the left optic nerve, with increased T2 hyperintensity in the perineural space and enhancement post contrast (Figure 2). These features are suggestive of optic neuritis. There is no evidence of intracranial or intraspinal lesions.

**Figure 2:**
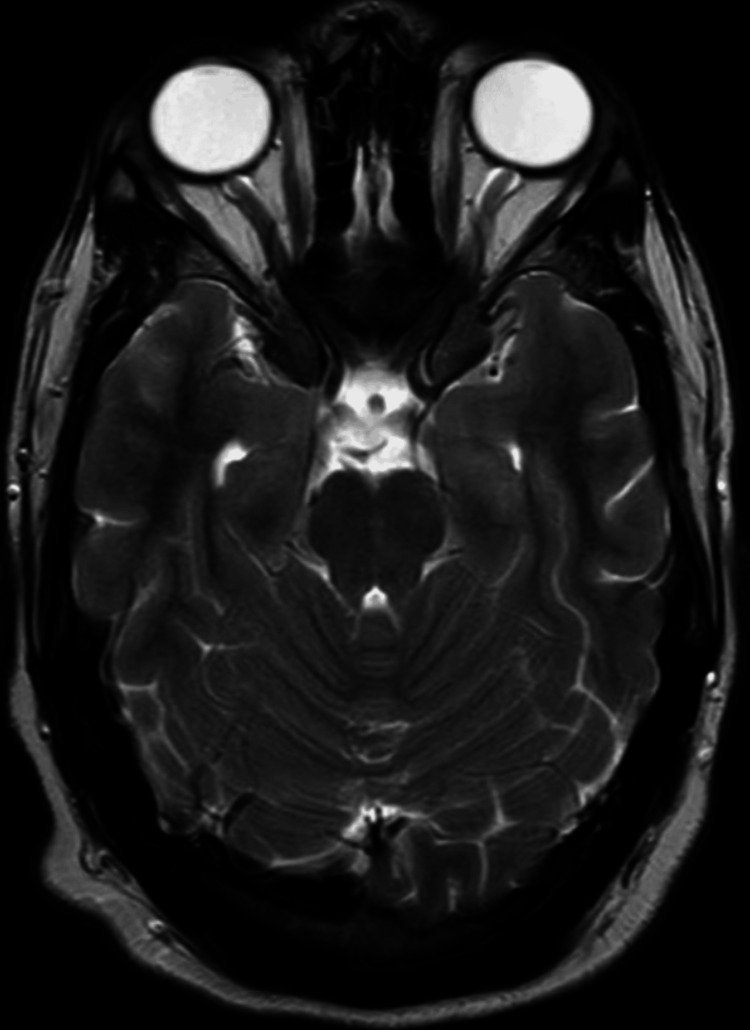
Axial T2-weighted MRI of the brain and orbits showing a mildly enlarged intraorbital segment of the left optic nerve with increased hyperintense cerebrospinal fluid (CSF) signal in the perineural space.

The patient was co-managed with the infectious disease team. He was started on anti-TB treatment, which was Akurit-4, and concurrently prescribed pyridoxine. He responded well to the anti-TB treatment. A month after treatment, aided VA for both eyes was 6/6 with normal red and light saturation and a normal Ishihara test. The optic disc swelling and central scotoma resolved (Figures 3A-3B). Anti-TB treatment was continued with close monitoring in the eye clinic.

**Figure 3:**
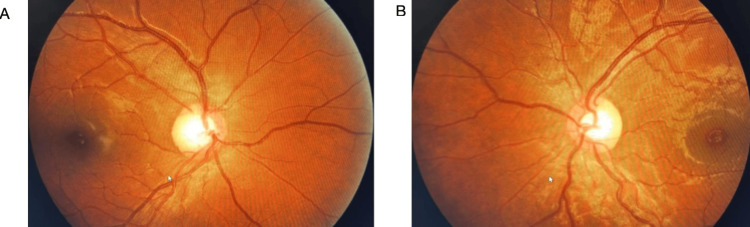
(a) Normal right eye fundus. (b) Resolved left optic disc hyperaemia with a well-defined disc margin.

## Discussion

Ocular TB can affect any part of the globe, orbital contents, optic nerve, and the orbital apex. It has a wide spectrum of clinical presentation, with uveitis being the most common [5,6]. Other than that, it also presents as conjunctival abscess, subconjunctival granuloma, episcleritis, scleritis, corneal ulcer, retinitis, vasculitis, choroiditis, choroidal granuloma, optic neuropathy, orbital apex syndrome, orbital cellulitis, endophthalmitis, and even panophthalmitis [5,6].

Optic neuropathy can present as optic perineuritis, optic neuritis, optic nerve tubercle, papilloedema, papillitis, retrobulbar neuritis, neuroretinitis, compressive optic neuropathy, and anterior ischemic optic neuropathy [5-9]. Among these, papillitis is the most commonly reported form of optic neuropathy [5,7,8].

The pathophysiology of TB-associated optic neuritis involves both direct and indirect mechanisms. Directly, there can be haematogenous spread of MTB to the eye, causing inflammation of the optic nerve sheath, compression, and impairment of optic nerve function [6,9]. Furthermore, tuberculomas can form in the brain and directly invade the optic nerve or cause localised inflammation, disrupting the optic nerve function [9]. The indirect mechanism involves an inflammatory response to the systemic TB infection, leading to secondary effects on the optic nerve. Additionally, severe TB is associated with impaired vascular conditions, which may cause ischemic optic nerve damage. In the presence of TB meningitis, raised intracranial pressure can also affect the optic nerve [9].

Common ocular symptoms in TB optic neuritis are reduced vision and visual field defects, such as central scotoma or peripheral field defects. Some may also present with eye pain, especially on eye movement, eye redness, impaired colour vision, diplopia, ptosis, and floaters. Fundus examination may show disc oedema; however, in some cases, the optic disc may be normal [9].

Diagnosing TB continues to be a challenge to ophthalmologist as patients do not always present with typical systemic symptoms suggestive of TB. Besides that, diagnostic criteria have not been designated for tuberculous optic neuropathy, and difficulty in obtaining microbiological evidence or biopsy of the intraocular tissue makes diagnosis even more difficult for ophthalmologists [5,7]. Procedures such as vitreous tap, aqueous paracentesis, or retinal biopsy are not done worldwide due to low sample volume obtained and the possibility of worsening inflammation after the procedures [5].

Our patient presented with clinical features of optic neuritis without any systemic manifestation of TB. However, our suspicion for TB was high as he was an inmate living in a crowded prison with a raised ESR, positive Mantoux test, and positive TB Quantiferon. His MRI was also suggestive of optic neuritis. To further support the diagnosis of TB optic neuritis, this patient responded well to anti-TB treatment as evidenced by improving visual acuity from hand movement to 6/6, normal colour vision, and resolved optic disc swelling.

Management involves treating the TB infection with antitubercular medication. According to the fourth edition of clinical practice guidelines for TB management, the recommended treatment for other forms of extrapulmonary TB, except bone, joint, and meningitis, is the 2EHRZ/4HR regimen (the initial two months of intensive phase includes treatment with ethambutol, isoniazid, rifampicin, and pyrazinamide, and subsequently continued with four months of isoniazid and rifampicin) [10].

According to Davis et al., there was no medication-induced optic neuropathy in patients receiving ethambutol. This answers the dilemma regarding starting ethambutol as a first-line anti-TB drug in managing TB optic neuritis [7]. However, the patient's optic nerve function, such VA, colour vision, and visual field, should be closely monitored for any evidence of drug toxicity.

## Conclusions

Ocular TB can present with various clinical symptoms and signs, which may mimic other diseases. Posterior segment involvement in ocular TB is highly associated with significant visual impairment. A high index of suspicion is required for the diagnosis of TB-associated optic neuritis, as corticosteroids, which are the standard treatment for optic neuritis, may be sight- and even life-threatening if used without initiation of anti-tubercular therapy. Early and prompt treatment is important to improve outcomes and prevent visual loss.
